# Neuroendoscopic single-nostril nasoseptal approach for tuberculum sellae meningiomas: technical description and preliminary clinical experience

**DOI:** 10.3389/fneur.2026.1889000

**Published:** 2026-08-05

**Authors:** Jinshen Xie, Tiejian Liu, Lei Gao, Haijun Xia, Zhenhua Song, Hongshen Zhu, Yuping Peng

**Affiliations:** 1 Department of Neurosurgery, The Third Affiliated Hospital of Southern Medical University, Guangzhou, Guangdong, China; 2 Department of Neurosurgery, Nanfang Hospital Southern Medical University, Guangzhou, Guangdong, China

**Keywords:** nasoseptal approach, neuroendoscopy, single-nostril approach, skull base surgery, tuberculum sellae meningioma

## Abstract

**Objective:**

To describe the surgical technique and preliminary feasibility of neuroendoscopic resection of selected tuberculum sellae meningiomas through a single-nostril nasoseptal approach.

**Methods:**

Two female patients with small, centrally located tuberculum sellae meningiomas (13 × 11 × 10 mm and 16 × 11 × 12 mm) underwent tumor resection through a single-nostril nasoseptal approach. Neither tumor showed marked lateral extension, optic canal invasion, cavernous sinus involvement, or vascular encasement. A transseptal submucosal corridor was created through one nostril. A rigid nasal retractor was used in the first patient, whereas a soft-channel system was used in the second. Multilayer skull base reconstruction was performed using artificial materials.

**Results:**

Both procedures were completed through the planned unilateral corridor without conversion to a binostril or transcranial approach. Complete tumor removal was achieved in both patients based on intraoperative endoscopic inspection and early postoperative MRI. Pathology confirmed WHO grade 1 meningioma in both cases. Both patients reported postoperative visual improvement, and pituitary function remained normal without diabetes insipidus. The first patient developed transient postoperative cerebrospinal fluid rhinorrhea, which resolved with conservative management. The second patient had no postoperative cerebrospinal fluid leakage. Follow-up nasal endoscopy showed satisfactory mucosal healing without septal perforation, nasal adhesion, or apparent anatomical deformity.

**Conclusion:**

The single-nostril nasoseptal approach may provide a feasible complementary endonasal corridor for carefully selected small, centrally located tuberculum sellae meningiomas. The soft-channel system may improve instrument maneuverability within the narrow operative space. However, this preliminary two-patient experience cannot establish the safety, efficacy, reproducibility, or superiority of this approach. Larger studies with longer follow-up and standardized functional assessments are needed.

## 1 Introduction

Tuberculum sellae meningiomas (TSMs) arise from the junction of the tuberculum sellae and the planum sphenoidale and are closely related to the optic nerves and chiasm, internal carotid arteries, anterior cerebral artery–anterior communicating artery complex, pituitary stalk, and superior hypophyseal arteries (1). These anatomical relationships make preservation of visual, vascular, and pituitary function central to surgical management. Various transcranial approaches, including the pterional, subfrontal, and supraorbital approaches, have been developed for TSM resection, with the choice of approach largely depending on tumor anatomy and extension (2–5). Transcranial approaches remain important, particularly for tumors with marked lateral extension, vascular encasement, or anatomical relationships that are unfavorable for an endonasal route. In contrast, the expanded endoscopic endonasal approach (EEA) provides a direct ventral midline trajectory to the tuberculum sellae, allowing early access to the tumor attachment and inferior visualization of the optic nerves and chiasm (6–8). Therefore, transcranial and endonasal approaches should be regarded as complementary rather than competing strategies. Surgical approach selection should be individualized according to tumor size, lateral extension, optic canal involvement, vascular relationships, sinonasal anatomy, and the experience of the surgical team (9, 10).

The binostril expanded EEA is an important surgical option for selected TSMs and provides sufficient instrument separation for two-surgeon, four-handed microsurgical dissection. However, contemporary endoscopic skull base surgery does not follow a uniform pattern of sinonasal exposure or reconstruction. The middle turbinates may be lateralized and preserved, the extent of posterior septectomy can be tailored to the required surgical corridor, and the use of vascularized nasoseptal flaps or autologous grafts should be individualized according to the size of the osteodural defect, the characteristics of intraoperative cerebrospinal fluid leakage, previous surgical history, and patient-specific risk factors (11). Thus, middle turbinate resection, extensive posterior septectomy, pedicled nasoseptal flap harvest, and autologous tissue grafting are not mandatory components of every expanded EEA. Nevertheless, in appropriately selected cases, limiting manipulation of the nasal corridor while maintaining adequate visualization and instrument maneuverability remains a relevant technical objective.

Single-nostril endoscopic approaches have been used primarily for pituitary and other sellar lesions. A unilateral nasal corridor may reduce manipulation of bilateral sinonasal structures, but the limited working space may increase instrument crowding and restrict operative freedom, particularly during delicate suprasellar dissection. Our team has accumulated extensive experience with the single-nostril transnasoseptal corridor in pituitary surgery. Building on this experience, we applied this corridor to two carefully selected small, centrally located TSMs. Neither patient had optic canal invasion, marked lateral extension, or vascular encasement, and both had well-pneumatized sphenoid sinuses. In this technical report, we describe the creation of the single-nostril nasoseptal corridor, the use of specialized instruments, the role of a soft-channel system in improving maneuverability within a narrow working space, and multilayer skull base reconstruction using artificial materials. The purpose of this report is to demonstrate the technical feasibility and operative nuances of this approach in carefully selected patients. Rather than replacing contemporary binostril expanded EEA or transcranial approaches, the single-nostril nasoseptal approach may serve as a complementary endonasal strategy for selected small, centrally located TSMs.

## 2 Methods

### 2.1 Patient selection

This retrospective technical report included two female patients, aged 51 and 61 years, with tuberculum sellae meningiomas who underwent neuroendoscopic tumor resection through a single-nostril nasoseptal approach. Preoperative contrast-enhanced MRI showed tumor dimensions of 13 × 11 × 10 mm in Patient 1 and 16 × 11 × 12 mm in Patient 2. The surgical approach was selected after assessment of tumor location, lateral extension, optic canal involvement, relationships with adjacent neurovascular structures, and sinonasal anatomy. In both patients, the tumors were centrally located at the tuberculum sellae, without optic canal invasion, cavernous sinus involvement, marked extension beyond the lateral margins of the optic nerves or internal carotid arteries, or radiological evidence of vascular encasement. The tumors were in close contact with adjacent vessels, but the vessel lumens and walls remained intact. Both patients had well-pneumatized sphenoid sinuses and nasal anatomy suitable for creation of a transseptal submucosal corridor. These anatomical features represent the selection characteristics of the present two cases rather than definitive indications for this approach. The small sample size does not allow determination of a maximum tumor size or broader indication range for this technique. Preoperative evaluation included neurological examination, formal ophthalmological assessment with visual acuity and visual field testing, contrast-enhanced sellar MRI, skull base and paranasal sinus CT, vascular imaging, and pituitary function assessment. Preoperative pituitary function was normal in both patients.

### 2.2 Equipment and instrument preparation

**Figure 1:**
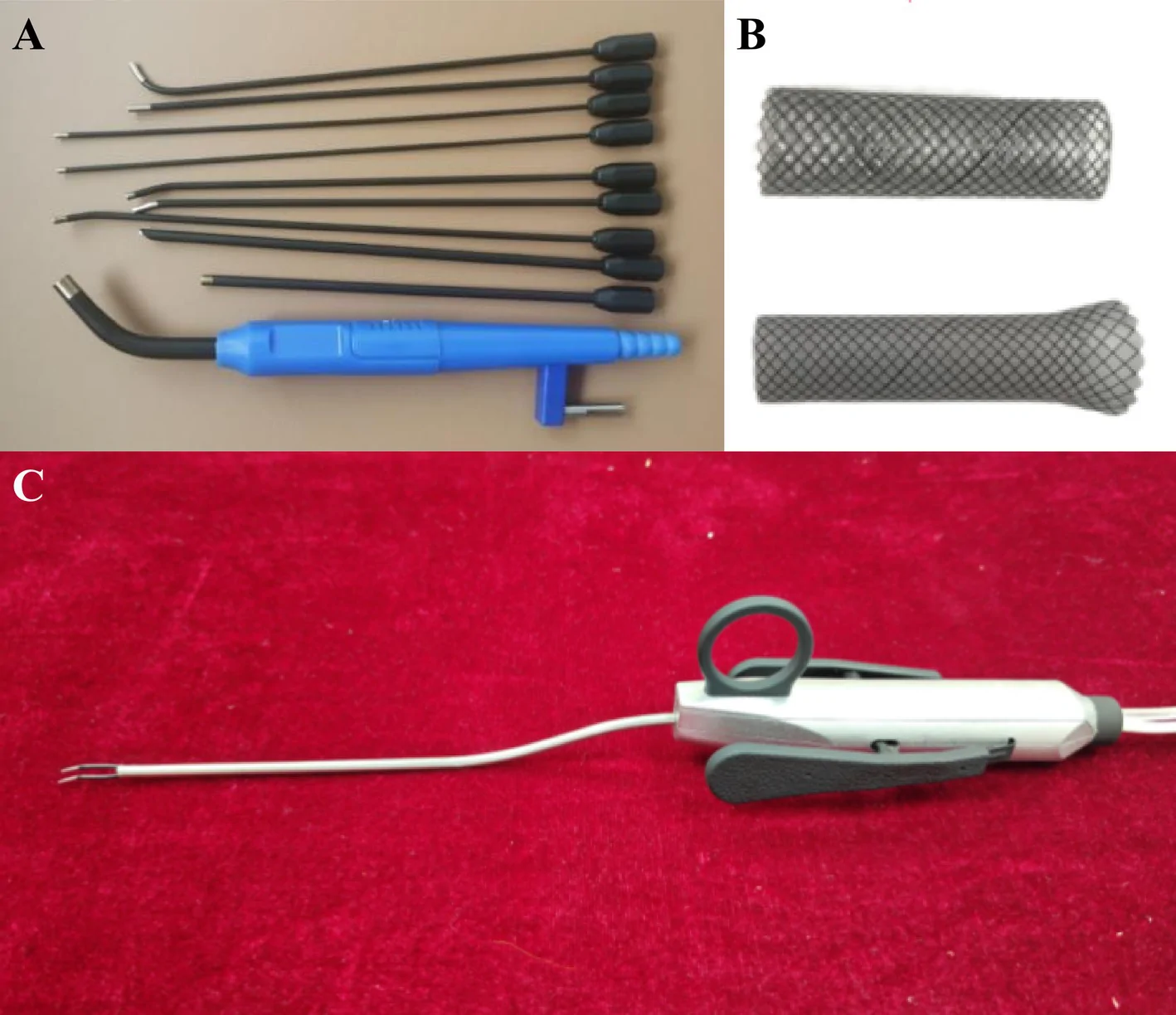
Surgical instruments used in the single-nostril nasoseptal approach. **(A)** Multifunctional angled suction device. **(B)** Soft-channel system. **(C)** Rifle-shaped single-tube bipolar electrocoagulator.

A rigid 0° neuroendoscope was used for the principal surgical steps, and a 30° neuroendoscope was used when angled visualization of the suprasellar region, parasellar region, or surgical margins was required. The endoscopic systems consisted of an IXON 3D or 4 K rigid neuroendoscope with the corresponding monitoring system. Microsurgical instruments included bayoneted or angled dissectors, curettes, scissors, grasping forceps, and tumor forceps. A multifunctional angled suction-coagulation device [Peng’s multifunctional operative dissector, Zhejiang Shuyou Technology Co., Ltd., Zhejiang, China] was used for suction, dissection, and focal coagulation (Figure 1A). A rifle-shaped single-tube bipolar coagulator [Disposable Single Tube Ablation Electrode, Zhenjiang Hengsheng Juanen Medical Instrument Co., Ltd. Jiangsu, China] was used for bipolar hemostasis within the restricted operative corridor (Figure 1C). A rigid nasal retractor [Nasal retractor, Hunan Jingsheng Medical Technology Co., Ltd., Hunan, China] was used in Patient 1. The rigid retractor provided stable separation of the bilateral septal mucosal layers, but it restricted lateral and vertical instrument movement within the narrow single-nostril corridor. Therefore, in Patient 2, after creation of the transseptal submucosal corridor and exposure of the anterior wall of the sphenoid sinus, a soft-channel system [Disposable sterile transnasal support catheter; Micro-Tech Nuonuo Medical Technology (Nanjing) Co., Ltd., Nanjing, China] was used instead (Figure 1B). The soft channel maintained separation between the bilateral septal mucosal layers while allowing greater instrument mobility than the rigid retractor. Its compliant wall also reduced direct friction between repeatedly introduced surgical instruments and the septal mucosa.

### 2.3 Surgical technique

**Figure 2:**
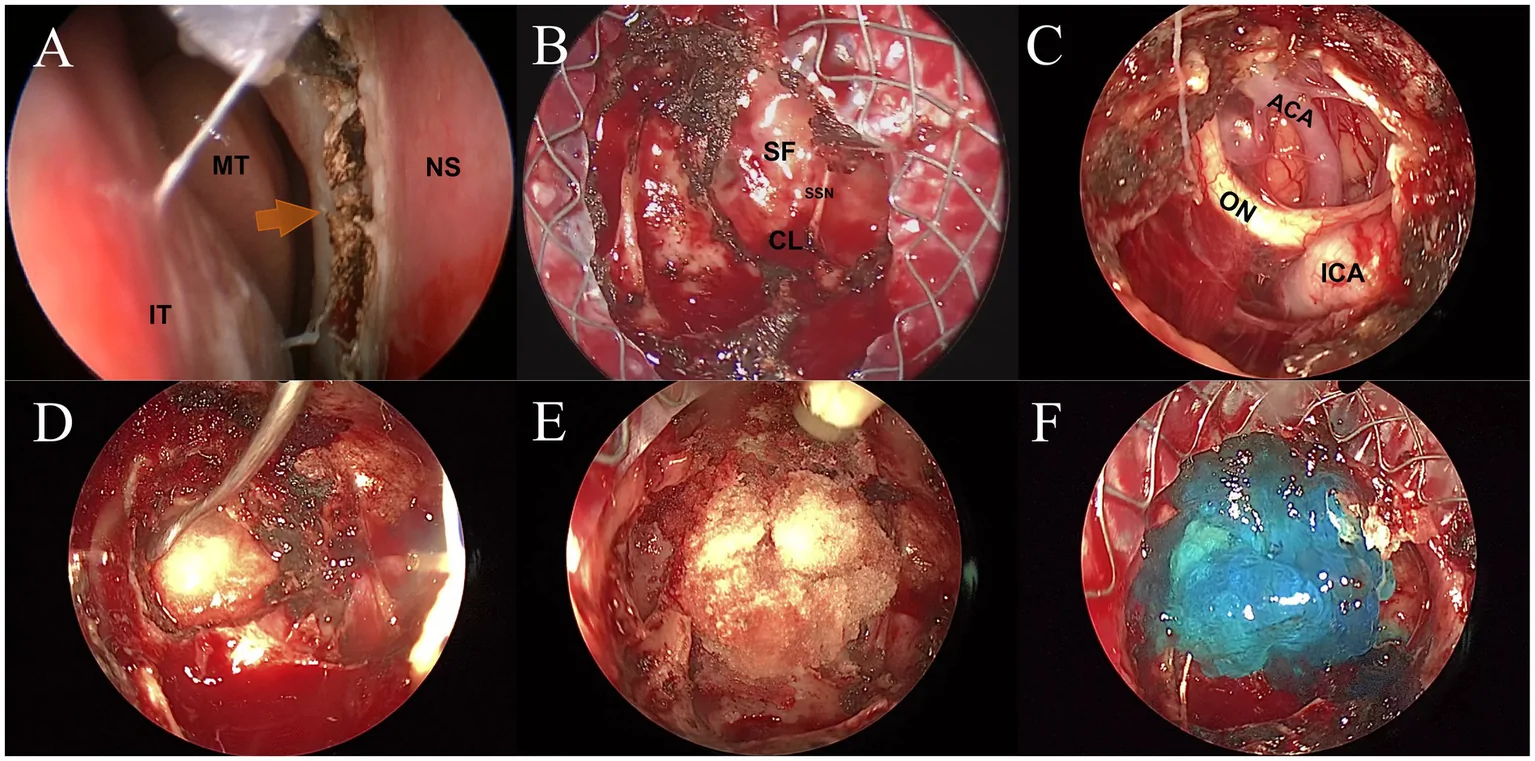
Intraoperative endoscopic views. Nasal septal mucosal incision **(A)**. Exposure of sellar floor structures **(B)**. Visualization of neurovascular structures after tumor debulking **(C)**. Artificial dural graft for skull base reconstruction **(D)**. Gelatin sponge for reinforcement **(E)**. Fibrin glue sealing the skull base defect **(F)**. IT, inferior turbinate; MT, middle turbinate; NS, nasal septum; SF, sellar floor; CL, clivus; SSN, suprasellar notch; ACA, anterior cerebral artery; ON, optic nerve; ICA, internal carotid artery.

After induction of general anesthesia and endotracheal intubation, the patient was placed in the supine position with the upper body elevated by approximately 30° and the head rotated approximately 20° to the right. The surgeon stood on the patient’s right side. The face and bilateral nasal cavities were disinfected and irrigated with diluted povidone-iodine solution. The right nasal cavity was temporarily packed with epinephrine-soaked cottonoids to promote mucosal decongestion and enlarge the operative corridor. A 0° neuroendoscope was introduced through the right nostril. The septal tubercle was used as the principal anatomical landmark. A longitudinal incision approximately 1.5 cm in length was made in the septal mucosa at or immediately anterior to the septal tubercle. The precise incision site was planned according to the anticipated skull base defect and reconstruction requirements. When a relatively large osteodural defect or a higher risk of reconstruction failure was anticipated, the septal incision could be designed more anteriorly, that is, as an anterior septal incision, to allow extension of the incision and preparation of a pedicled nasoseptal flap if necessary. This contingency design was used in Patient 1. Preservation of future reconstructive options requires maintenance of septal mucosal integrity and protection of the posterior septal vascular pedicle. The mucoperichondrial and mucoperiosteal planes were dissected between the bilateral septal mucosae and the cartilaginous or bony septum. A limited portion of the bony septum was removed to create a controlled transseptal corridor. Care was taken to preserve the bilateral septal mucosal layers and avoid unnecessary posterior septectomy. A rigid nasal retractor was placed between the bilateral septal mucosae in Patient 1. In Patient 2, after exposure of the anterior wall of the sphenoid sinus, the soft-channel system was inserted to support the mucosal corridor and improve instrument maneuverability. The anterior wall of the sphenoid sinus was opened under direct endoscopic visualization. The extent of sphenoidotomy was tailored to the exposure required for each tumor. The sphenoid sinus mucosa overlying the planned skull base opening was removed, exposing the sellar floor, tuberculum sellae, adjacent planum sphenoidale, carotid protuberances, and medial optocarotid recesses. A high-speed drill was used to remove the bone overlying the tumor attachment. The approximate dimensions of the bony opening were 1.5 × 1.5 cm in Patient 1 and 2 × 2 cm in Patient 2. Before dural opening, the dural attachment and tumor base were coagulated to reduce tumor blood supply. The dura was then opened under direct visualization, resulting in high-flow cerebrospinal fluid egress. Before tumor mobilization, the relationship of the tumor to the optic nerves and chiasm, internal carotid arteries, anterior cerebral artery–anterior communicating artery complex, and pituitary stalk was assessed. Feeding vessels arising from the dural attachment and tumor base were coagulated and divided early when they could be safely identified. The tumor was then separated from the surrounding arachnoid planes using a combination of blunt and sharp dissection. Sharp dissection was used for focal adhesions to avoid traction on the optic nerves, optic chiasm, or adjacent vessels. Within the restricted corridor, limited internal debulking was performed when additional working space was required. The tumor margins were progressively and circumferentially mobilized. The tumor was then slowly rotated to confirm that no residual adhesions remained. Both tumors were in close contact with adjacent vessels but did not encase them. The arachnoid planes around the optic nerves, internal carotid arteries, and anterior cerebral artery complex were preserved whenever identifiable. The pituitary stalk was identified and protected. Both tumors were completely removed under direct endoscopic visualization, and early postoperative contrast-enhanced MRI confirmed no obvious residual tumor. After tumor removal, the dural attachment was further coagulated. The operative field was inspected using 0° and 30° endoscopes, with particular attention to the optic nerves and chiasm, pituitary stalk, adjacent vessels, tumor bed, and margins of the bony opening. Multilayer skull base reconstruction was performed using commercially available non-autologous materials in both patients. No abdominal fat, fascia lata, free mucosal graft, or pedicled nasoseptal flap was harvested. The first layer consisted of an intradural collagen-based dural substitute [collagen-based artificial dura mater; Tianxinfu (Beijing) Medical Appliance Co., Ltd., Beijing, China], which was placed beneath the dural margins and secured using tissue adhesive [Glubran® 2 surgical glue; GEM S.r.l., Viareggio, Italy]. A second biological dural graft [biological dural patch; Guanhao Biotech Co., Ltd., Guangdong, China] was placed as an onlay layer over the osteodural defect. Gelatin-based material [Absorbable gelatin sponge; Jiangxi Xiang’en Medical Technology Development Co., Ltd., Jiangxi, China] was then placed around the graft margins to reinforce the peripheral seal. Finally, fibrin sealant [SainaoNing® absorbable dural sealant; Success Bio-Tech Co., Ltd., Jinan, Shandong, China] was applied over the entire reconstructed area. The size and amount of each reconstructive material were adjusted according to the extent of the skull base defect. If secure closure could not be achieved with this strategy, conversion to fascia lata grafting or pedicled nasoseptal flap reconstruction was considered as an available alternative. After reconstruction, the rigid retractor or soft-channel system was removed. The bilateral septal mucosal layers were repositioned. Expandable nasal packing [Merocel® hemoX high-expansion hemostatic material; Medtronic, Shanghai, China] was placed in the nasal cavities to support mucosal apposition and was removed on postoperative day 2 (Figure 2).

### 2.4 Postoperative evaluation and follow-up

Postoperative neurological evaluation included assessment of consciousness, pupillary responses, ocular movements, visual symptoms, motor function, and evidence of cerebrospinal fluid rhinorrhea. Urine output, serum electrolytes, and pituitary hormone levels were monitored to detect diabetes insipidus or postoperative pituitary dysfunction. Early postoperative contrast-enhanced MRI was performed to evaluate the extent of tumor resection and identify postoperative complications. Subsequent MRI was used to assess tumor recurrence. Postoperative visual improvement was assessed according to patient-reported symptoms and bedside confrontation visual field testing. Nasal endoscopy was performed during follow-up to evaluate septal mucosal healing, crusting, adhesions, septal perforation, anatomical deformity, evidence of cerebrospinal fluid leakage, and the condition of the posterior nasal cavity. Patient 1 underwent nasal endoscopic assessment at 12 months, and Patient 2 underwent nasal endoscopic assessment at 30 days. Standardized olfactory testing and validated sinonasal quality-of-life questionnaires were not prospectively administered. Therefore, sinonasal outcomes were assessed mainly according to clinical symptoms and nasal endoscopic findings.

## 3 Results

Two female patients, aged 51 and 61 years, underwent neuroendoscopic resection of tuberculum sellae meningiomas through the single-nostril nasoseptal approach. Both procedures were completed through the planned unilateral corridor without conversion to a binostril or transcranial approach. A rigid nasal retractor was used in Patient 1, whereas the soft-channel system was used in Patient 2. The approximate dimensions of the skull base openings were 1.5 × 1.5 cm and 2 × 2 cm, respectively. Complete tumor removal was achieved in both patients based on intraoperative endoscopic inspection and early postoperative contrast-enhanced MRI. Histopathological examination demonstrated a psammomatous meningioma, WHO grade 1, in Patient 1 and a fibrous meningioma, WHO grade 1, in Patient 2. Both patients reported improvement in their preoperative visual symptoms, and bedside confrontation testing showed improvement in the preoperative visual field abnormalities. Preoperative and postoperative pituitary function was normal in both patients, and neither patient developed diabetes insipidus or clinically evident postoperative hypopituitarism. Nasal packing was removed on postoperative day 2 in both patients. Patient 1 developed transient postoperative cerebrospinal fluid rhinorrhea, which resolved after 3 weeks of conservative management, including strict bed rest, without surgical revision. Patient 2 had no postoperative cerebrospinal fluid leakage. Follow-up nasal endoscopy demonstrated satisfactory healing of the septal mucosa without septal perforation, nasal adhesion, or apparent anatomical deformity. Neither patient reported clinically apparent deterioration in olfactory function. At 12 months after surgery, MRI in Patient 1 showed no evidence of residual or recurrent tumor, and nasal endoscopy demonstrated preservation of the nasal anatomy. Follow-up MRI in Patient 2 showed no obvious residual tumor or other abnormal findings, and nasal endoscopy at 30 days demonstrated an intact posterior nasal cavity without apparent septal deformity or mucosal defect. Surgical videos with narration were recorded for both procedures (Supplementary Video S1; in Patient 1 Supplementary Video S2) in Patient 2.

### 3.1 Patient-level clinical details

#### 3.1.1 Patient 1 (Supplementary Video S1)

**Figure 3:**
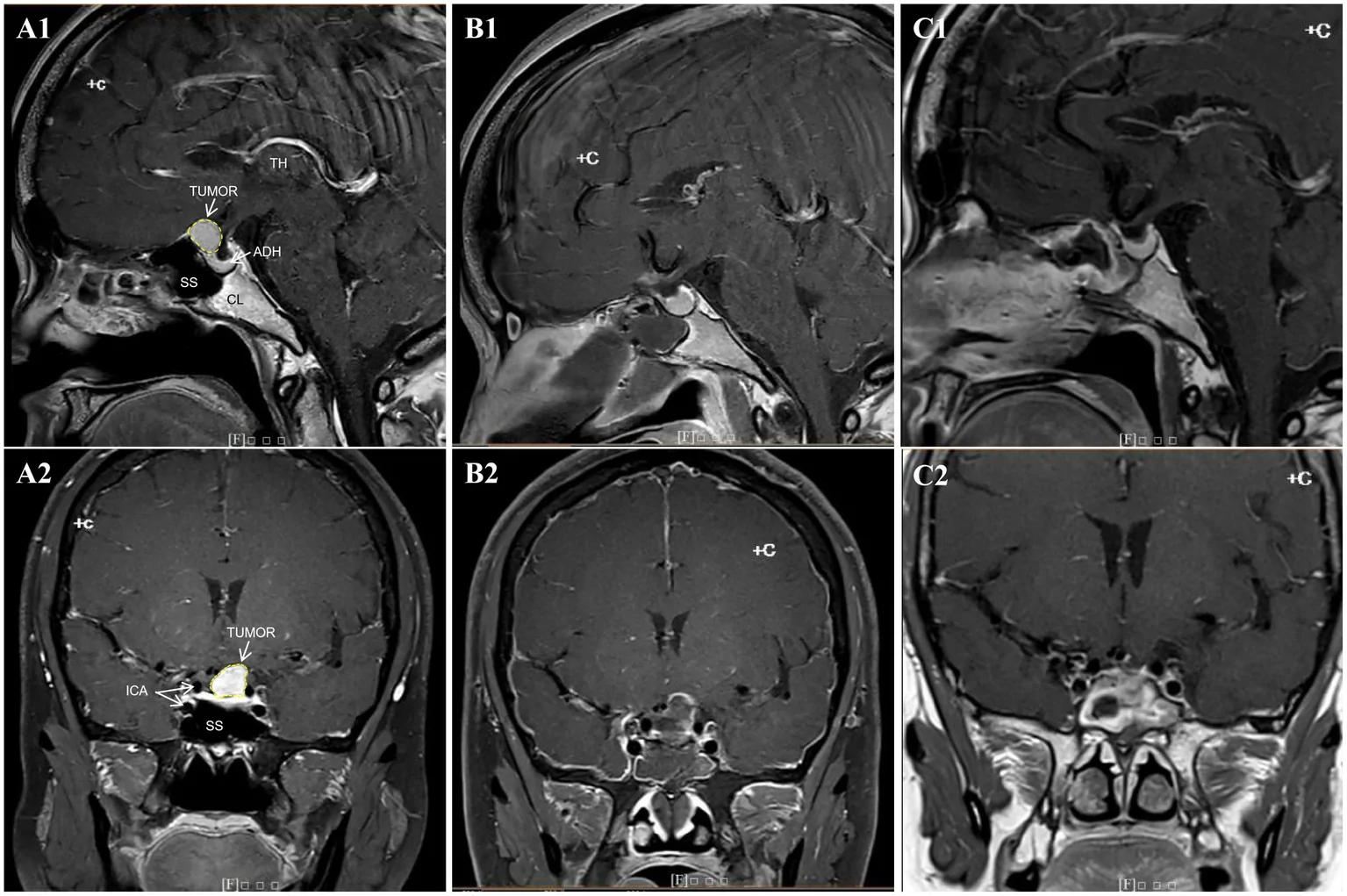
Preoperative, postoperative, and 12-month follow-up magnetic resonance images (MRI) of Patient 1. **(A)** Preoperative sagittal and coronal contrast-enhanced MRI. **(B)** Postoperative sagittal and coronal MRI confirming gross total resection. **(C)** Twelve-month follow-up MRI showing no tumor recurrence. TH, thalamus; ADH, adenohypophysis; SS, sphenoid sinus; CL, clivus; ICA, internal carotid artery.

A 51-year-old woman presented with a history of headache for more than 6 months and progressively blurred vision. Formal preoperative ophthalmological examination showed uncorrected visual acuity of 0.2 in the right eye and 0.2 − in the left eye, with corrected visual acuity of 1.0 in both eyes. Visual field testing demonstrated an irregular inferonasal scotoma in the right eye and peripheral visual field defects in the left eye. Contrast-enhanced MRI demonstrated a 13 × 11 × 10 mm tuberculum sellae meningioma. The tumor was centrally located, without optic canal invasion, marked lateral extension, or vascular encasement. The sphenoid sinus was well pneumatized (Figure 3). A rigid nasal retractor was used to maintain the transseptal operative corridor. The tumor was circumferentially separated from the surrounding arachnoid planes and completely removed under endoscopic visualization. Early postoperative contrast-enhanced MRI showed no obvious residual tumor. Histopathological examination demonstrated a psammomatous meningioma, WHO grade 1. Postoperatively, the patient reported visual improvement, and bedside confrontation testing showed improvement in the preoperative visual field abnormalities. Pituitary function remained normal, and no diabetes insipidus occurred. The patient developed transient cerebrospinal fluid rhinorrhea, which resolved after 3 weeks of strict bed rest and conservative treatment without revision surgery. At the 12-month follow-up, MRI showed no tumor recurrence, and nasal endoscopy demonstrated healed septal mucosa without septal perforation or apparent anatomical deformity.

#### 3.1.2 Patient 2 (Supplementary Video S2)

**Figure 4:**
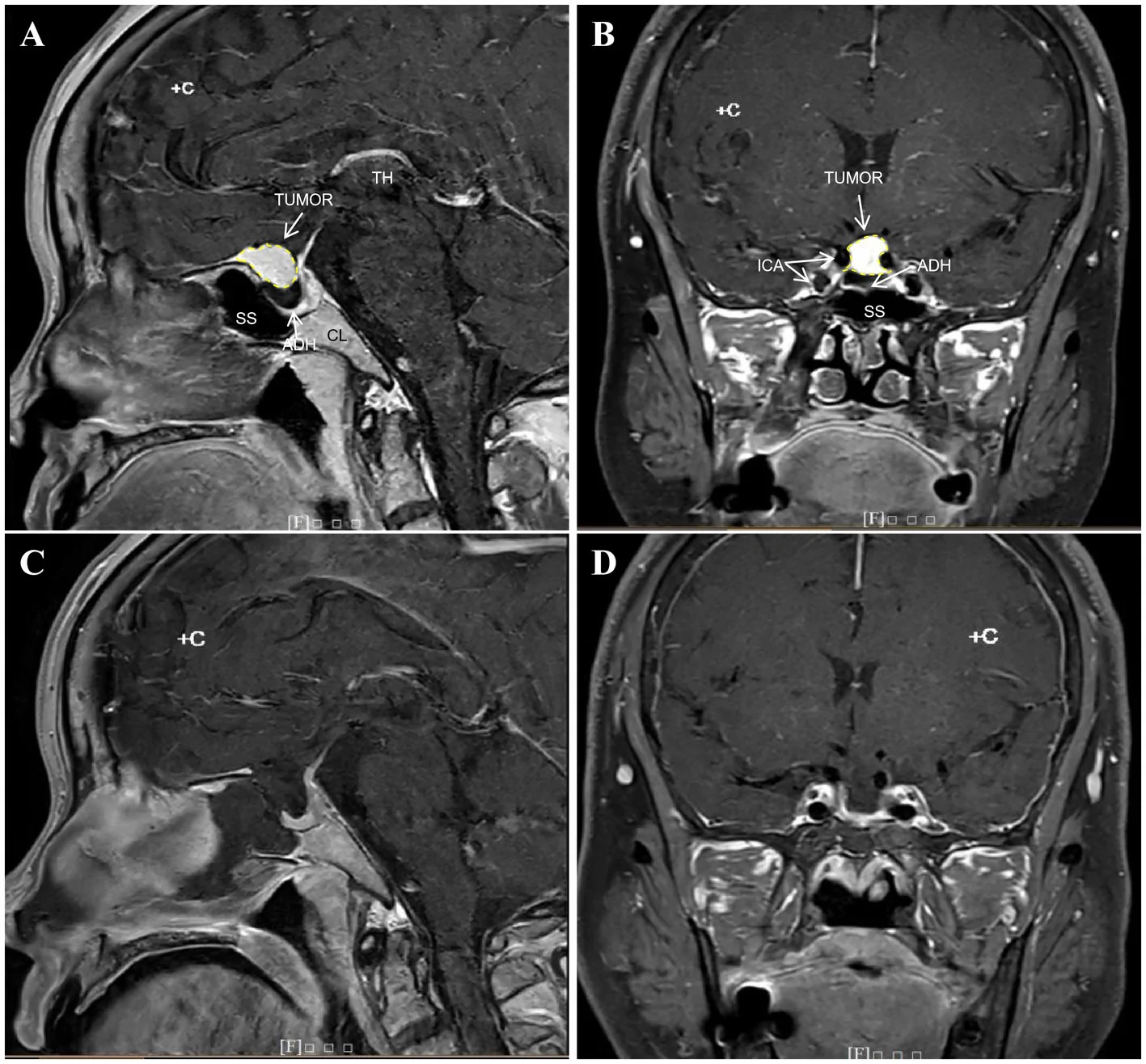
Preoperative and postoperative MRI of Patient 2. **(A,B)** Preoperative sagittal and coronal images demonstrating a Tuberculum Sellae meningioma. **(C,D)** Postoperative images showing complete tumor removal. TH, thalamus; ADH, adenohypophysis; SS, sphenoid sinus; CL, clivus; ICA, internal carotid artery.

**Figure 5:**
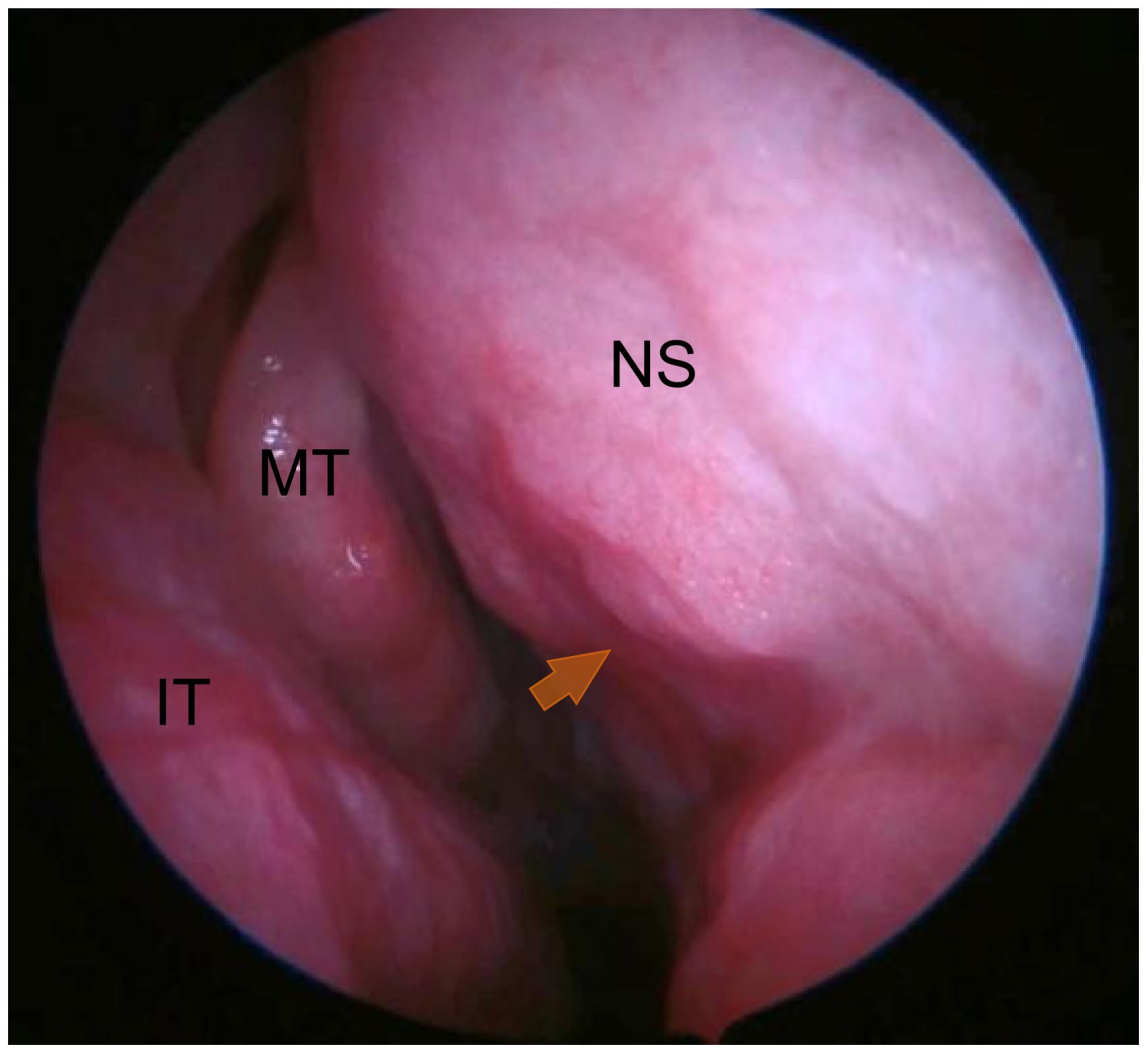
Postoperative nasal endoscopic view at 30 days in Patient 2, showing intact nasal mucosa and normal anatomy without scarring or deformity. The yellow arrow indicates the original incision. IT, inferior turbinate; MT, middle turbinate; NS, nasal septum.

A 61-year-old woman presented with recurrent frontal and temporal headache and dizziness for 7 months, followed by worsening symptoms with blurred vision and limb numbness before admission. Formal preoperative ophthalmological examination showed uncorrected visual acuity of 0.6 in the right eye and 0.8 in the left eye, with corrected visual acuity of 1.0 in both eyes. The visual field was normal in the right eye, whereas superior and nasal peripheral visual field defects were identified in the left eye. Contrast-enhanced MRI demonstrated a 16 × 11 × 12 mm tuberculum sellae meningioma. The tumor was centrally located, without optic canal invasion, marked lateral extension, or vascular encasement. The sphenoid sinus was well pneumatized (Figure 4). After creation of the transseptal submucosal corridor and exposure of the anterior sphenoid wall, the soft-channel system was inserted instead of a rigid retractor. The soft channel maintained the mucosal corridor while allowing greater instrument movement during the intradural procedure. The tumor was completely removed under endoscopic visualization, and early postoperative contrast-enhanced MRI showed no obvious residual tumor. Histopathological examination demonstrated a fibrous meningioma, WHO grade 1. The patient reported postoperative improvement in blurred vision, and bedside confrontation testing showed improvement in the preoperative left visual field abnormality. Pituitary function remained normal, and neither diabetes insipidus nor postoperative cerebrospinal fluid leakage occurred. Nasal endoscopy at 30 days showed satisfactory mucosal healing and an intact posterior nasal cavity without septal perforation or apparent anatomical deformity (Figure 5).

## 4 Discussion

Over the past two decades, advances in neuroendoscopy, high-definition imaging systems, specialized instrumentation, and skull base reconstruction have continuously shaped the surgical strategy for tuberculum sellae meningiomas (TSMs). Modern endoscopic systems provide close-up and wide-angle visualization of the tumor attachment, optic nerves and chiasm, pituitary stalk, internal carotid arteries, and anterior circulation (12). The use of three-dimensional endoscopy may further improve spatial perception and depth judgment in the complex anterior skull base, facilitating precise intraoperative localization and safe dissection. For TSMs, the selection of surgical approach should still be determined mainly by tumor size, lateral extension, optic canal involvement, vascular encasement, tumor consistency, degree of calcification, and surgeon experience. Transcranial approaches, including pterional, subfrontal, and supraorbital approaches, remain important for tumors with marked lateral extension, vascular encasement, or anatomical relationships unfavorable for endonasal surgery (2–5). In contrast, the expanded endoscopic endonasal approach (EEA) provides a direct ventral midline route to the tuberculum sellae, allowing early devascularization of the dural attachment and inferior visualization of the optic nerves and chiasm (6–8). Therefore, transcranial and endonasal approaches should be understood as complementary treatment strategies rather than mutually exclusive techniques (9, 10).

At present, the binostril expanded EEA is an important endonasal option for selected TSMs. This approach provides a relatively wide operative corridor, facilitates two-surgeon, four-handed cooperation, and allows multi-angled management of the tuberculum sellae, planum sphenoidale, medial optic canal, and suprasellar cistern (13, 14).However, contemporary expanded endonasal surgery is not a fixed procedure. Middle turbinate resection is not mandatory, and in many cases the middle turbinates can be lateralized and preserved. The extent of posterior septectomy, sphenoidotomy, and nasal mucosal manipulation should also be individualized according to lesion size, required exposure, and reconstruction strategy (11).Similarly, skull base reconstruction is not based on a single standardized method, but may incorporate different combinations of artificial materials, autologous fascia, fat, rigid buttress materials, pedicled nasoseptal flaps, and lumbar drainage according to the size of the osteodural defect, intraoperative CSF flow, previous surgical history, and patient-specific risk factors. Therefore, the present study does not position the single-nostril nasoseptal approach as a replacement for contemporary binostril expanded EEA, but rather as a complementary endonasal strategy that may be considered in carefully selected patients (Table 1).

**Table 1:** Technical comparison between the binostril expanded endoscopic endonasal approach and the single-nostril nasoseptal approach.

Surgical characteristics	Expanded endoscopic endonasal approach	Single_nostril nasoseptal approach
Incision site	Posterolateral nasal/sphenoid rostrum	Anterior nasal septum
Access road	Bilateral nostrils	Unilateral nostril (usually right)
Device configuration	Double three handed or four handed technique	Single-surgeon bimanual technique or two-surgeon three-hand technique
Working angle	Wider operating angle and vision	Relatively limited operating angle
Reconstruction material	Reconstruction is individualized and may include artificial materials, autologous grafts, rigid buttress, pedicled nasoseptal flap, and/or lumbar drainage	Pure artificial materials (dural substitute, gelatin sponge, biological glue)
Middle turbinate management	May be lateralized and preserved; resection is performed only when required for exposure	Preserved as an anatomical landmark only
Risk of nasal complications	Relatively high	Low
Postoperative nasal anatomy	Visible surgical trauma	Essentially intact with minimal discernible trauma

Single-nostril endoscopic techniques have traditionally been used mainly for pituitary and other sellar lesions. Their potential advantage lies in reducing manipulation of bilateral sinonasal structures; however, their limitations include a narrow working space, instrument interference, and restricted operative angles (15, 16).Previous reports on mononostril endoscopic transsphenoidal approaches for sellar lesions have suggested that this strategy is feasible in selected cases (15, 17). In contrast, the “one-and-a-half nostril” technique was developed to balance the advantages of mononostril and binostril approaches by increasing surgical freedom while reducing nasal tissue manipulation (18, 19).The single-nostril nasoseptal approach used in the present study differs from a simple unilateral transnasal route to the sphenoid sinus. Its core concept is to create a midline submucosal corridor through the nasal septum, using the septum as a reliable midline anatomical reference, while preserving the middle turbinate and most intranasal structures before entering the sphenoid sinus and tuberculum sellae region.

Our team has long applied the single-nostril nasoseptal approach in pituitary surgery and has compared the endoscopic transnasoseptal approach with the transsphenoidal anterior wall approach in previous anatomical and clinical studies (20–24). These studies suggested that the transnasoseptal corridor provides a direct midline trajectory and may reduce unnecessary manipulation of the nasal mucosa and intranasal structures in selected sellar lesions (25). The present study extends this experience to two TSMs. In both patients, the tumors were located in the midline or paramedian region, were relatively limited in size, and showed no marked lateral extension, optic canal invasion, cavernous sinus involvement, or major vascular encasement. Both patients also had well-pneumatized sphenoid sinuses. These features were important prerequisites for the application of this approach.

Case selection is the key prerequisite for safe implementation of the single-nostril nasoseptal approach. Based on our experience, this approach appears more suitable for patients in whom the main tumor bulk is located in the midline or near the midline, the tumor volume is relatively limited, lateral extension does not clearly exceed the supraclinoid internal carotid artery, major vessels are not extensively encased, and the main surgical manipulation is concentrated in the tuberculum sellae, planum sphenoidale, and medial optic canal region. Medial optic canal invasion should not be considered an absolute contraindication to this approach, because the ventral route allows direct access to the inferomedial wall of the optic canal. However, neither patient in the present study had optic canal invasion, and this issue requires further validation in additional cases. If the tumor clearly extends beyond the lateral margin of the internal carotid artery, grows circumferentially around the optic nerve, extensively encases the anterior communicating artery complex or perforating vessels, or shows severe calcification, firm consistency, or broad dural attachment, the single-nostril corridor may be insufficient for safe multi-angled dissection. In such cases, a binostril expanded EEA or a transcranial approach should be considered. Therefore, the single-nostril nasoseptal approach should be understood as an optimized strategy after strict case selection, rather than a universal substitute for binostril or transcranial surgery.

In addition to tumor-related factors, patient-specific sphenoid sinus and skull base anatomy should also be considered when selecting an endoscopic corridor. The degree and pattern of sphenoid sinus pneumatization, the relationship between the sellar floor, tuberculum sellae, planum sphenoidale, opticocarotid recesses, and carotid protuberances, as well as the nasal septal and intranasal anatomy, may influence the feasibility and safety of a limited single-nostril transseptal route. Although the study by Tozzi et al. focused on craniopharyngiomas rather than tuberculum sellae meningiomas, it highlighted the broader concept that patient-specific skull base anatomy can support surgical approach selection and help identify anatomical settings favorable for endoscopic endonasal surgery (26). In the present two patients, well-pneumatized sphenoid sinuses and favorable midline skull base anatomy facilitated exposure of the tuberculum sellae and adjacent planum through a limited transseptal corridor.

Several technical details are important for the implementation of this approach. In the present study, the septal tubercle was used as an important anatomical landmark. A longitudinal septal mucosal incision of approximately 1.5 cm, made anterior to or at the level of the septal tubercle, allowed entry into the submucosal plane and creation of a midline operative corridor. This method enables dissection along the natural septal plane, reduces extensive manipulation of bilateral nasal mucosa, and helps maintain intraoperative midline orientation. For cases in which a larger osteodural defect is anticipated or the need for nasoseptal flap reconstruction cannot be excluded preoperatively, a more anterior septal incision can be designed to preserve the possibility of extending the incision and harvesting a pedicled nasoseptal flap. This contingency design was used in Patient 1. It should be emphasized that preservation of the septal mucosa and posterior vascular pedicle is the prerequisite for maintaining future reconstructive options. Therefore, the incision site should be planned preoperatively according to tumor size, expected bony opening, and the reconstruction strategy.

After entering the sphenoid sinus, the extent of sphenoidotomy should not be mechanically minimized, but rather individualized according to tumor size and the exposure required. In the present study, the bony openings were approximately 1.5 × 1.5 cm and 2 × 2 cm, respectively. For TSMs, the sellar floor, tuberculum sellae, planum sphenoidale, carotid protuberances, and opticocarotid recesses should be adequately identified. Before dural opening, coagulation of the dural attachment and tumor base facilitates early reduction of tumor blood supply. During tumor dissection, the arachnoid plane should be followed as much as possible using a combination of blunt and sharp dissection. Traction should be avoided near the optic nerves, optic chiasm, internal carotid arteries, and anterior circulation, and sharp dissection should be used for focal adhesions. After circumferential mobilization of the tumor margin, the tumor can be slowly rotated to confirm that no residual adhesions remain before final extraction. After tumor removal, repeated inspection with 0° and 30° endoscopes is necessary to evaluate the optic nerves and chiasm, pituitary stalk, adjacent vessels, tumor bed, and margins of the bony opening, thereby reducing the risk of residual tumor.

Specialized instruments are essential for performing delicate procedures through this limited corridor. The multifunctional angled suction-coagulation device remains one of the most frequently used instruments in our practice (Figure 1A). By integrating suction, focal coagulation, and dissection, this instrument helps maintain a clear operative field, reduces the need for frequent instrument exchange, and facilitates incision, exposure, hemostasis, and dissection in a narrow space. Bayoneted or angled dissectors, curettes, grasping forceps, tumor forceps, and scissors reduce hand collision outside the nostril and help prevent the instrument shaft from obstructing the endoscopic view. The rifle-shaped single-tube bipolar coagulator, with its slender working end, allows hemostasis within a limited corridor while minimizing interference with visualization and other instruments. However, instrument refinement alone is only a prerequisite. Long-term coordination between the surgeon and assistant is equally important, because endoscope holding, suction, coagulation, dissection, and instrument exchange must all be performed in a stable and coordinated manner within a confined working space.

The soft-channel system used in Patient 2 is a technical refinement worth emphasizing. In Patient 1, a rigid retractor was used and provided stable separation of the bilateral septal mucosa, but its fixed structure restricted lateral and vertical instrument movement to some extent. In Patient 2, after the anterior wall of the sphenoid sinus had been exposed, the rigid retractor was replaced with a soft-channel system. The soft channel maintained the submucosal nasoseptal corridor while allowing greater instrument mobility during the intradural stage. Its compliant wall may also reduce friction between instruments and the septal mucosa during repeated instrument insertion and withdrawal. For the single-nostril nasoseptal approach, the value of the soft channel is not simply to enlarge the incision, but to improve instrument maneuverability and reduce mucosal compression within a limited corridor. Nevertheless, the soft channel should currently be regarded as a technical adjunct rather than evidence that sinonasal complications or clinical outcomes are improved. Its true value requires further validation in larger cohorts with standardized sinonasal outcome assessment and long-term follow-up.

Skull base reconstruction is critical for reducing postoperative CSF leakage after endoscopic endonasal surgery. In recent years, with the development of multilayer reconstruction, pedicled nasoseptal flaps, and selective lumbar drainage, the rate of postoperative CSF leakage has decreased substantially (27). Cavallo et al. described the 3F (Fat, Flap, and Flash) technique as an *in situ* multilayer reconstruction strategy that may be effective in selected cases after endoscopic endonasal skull base surgery (28). In addition, free graft-based techniques, such as the “Parachute” technique described by Fiore et al., have been proposed for the repair of high-flow anterior skull base CSF leaks (29). Some authors have proposed the temporoparietal fascia flap as an alternative reconstructive option in revision surgery when nasal mucosa is unavailable because of previous surgery or anatomical limitations (27, 30). In the present study, both patients underwent multilayer reconstruction using artificial materials alone: an intradural collagen-based artificial dura was placed as the first layer, a second biological membrane was placed over the bony window, and the outer layer was reinforced with gelatin sponge, surgical sealant, and fibrin glue (31, 32). This strategy avoided an additional harvesting incision and donor-site morbidity, while attempting to achieve multilayer closure without autologous tissue. In both patients, opening of the suprasellar cistern resulted in high-flow intraoperative CSF egress, and the bony openings measured approximately 1.5 × 1.5 cm and 2 × 2 cm, respectively. In both patients, high-flow intraoperative CSF leakage was controlled intraoperatively, suggesting that artificial materials may be considered as an option for reconstruction of small defects when meticulous technique is used. However, it should be emphasized that Patient 1 still developed postoperative low-flow intermittent CSF rhinorrhea, clinically presenting as clear nasal drainage of approximately one drop every 10 min, which resolved after conservative bed rest. Patient 2 did not develop postoperative CSF leakage. This indicates that the reliability of artificial-material-only reconstruction should be interpreted cautiously and should not be considered superior to autologous tissue or vascularized flap reconstruction.

Based on our experience, skull base reconstruction should be stratified according to the size of the bony opening, the extent of the dural defect, CSF flow, the degree of overlap of the reconstructive materials, and reconstruction tension. The above reports also support the principle that reconstruction should be individualized according to CSF leak characteristics, defect size, available reconstructive tissue, and the need for stable multilayer closure. For small defects in which sufficient overlap and stable support can be achieved, multilayer reconstruction using artificial materials alone may have the potential advantage of avoiding donor-site morbidity. However, for high-flow CSF leakage, especially when associated with larger dural defects, larger bony openings, revision surgery, or cases in which reconstruction tension is judged to be high intraoperatively, autologous fascia lata or a vascularized nasoseptal flap combined with artificial materials may be more reliable. The single-nostril nasoseptal approach emphasizes mucosal preservation, but this should not come at the expense of reconstruction reliability. If secure closure cannot be achieved using artificial materials alone, the reconstruction strategy should be adjusted promptly rather than adhering to a single repair method.

Postoperative surveillance is another important consideration for this approach. Because the single-nostril nasoseptal approach preserves more intranasal structures, the postoperative sinonasal anatomy differs from that after a standard binostril expanded EEA. Therefore, evaluation of the skull base reconstruction site should be based on a combination of clinical symptoms, nasal endoscopy, and imaging studies. Nasal endoscopy can be used to assess septal mucosal healing, crusting, adhesions, septal perforation, local inflammation, and suspicious fluid in the posterior nasal cavity (Figure 5). If the patient develops clear rhinorrhea, positional headache, fever, meningeal irritation, abnormal increase in nasal discharge, or new visual symptoms, MRI or CT should be performed to exclude CSF leakage, infection, pneumocephalus, graft displacement, or delayed skull base healing. Patients should also be informed about symptoms related to delayed CSF rhinorrhea and infection so that they can seek medical attention promptly.

In addition, the relatively limited sphenoidotomy used in this approach may theoretically affect long-term sphenoid sinus ventilation and drainage. Potential delayed complications include retained secretions, chronic sphenoid sinusitis, impaired mucociliary clearance, or delayed mucocele formation. In the two patients in this study, follow-up nasal endoscopy showed no septal perforation, nasal adhesion, or apparent anatomical deformity, and no clinically evident long-term sinonasal complications were observed during the available follow-up. However, these early findings are insufficient to exclude delayed sphenoid sinus complications. Longer endoscopic and radiological follow-up is needed to evaluate sphenoid sinus drainage, mucosal healing, and sinonasal outcomes.

### 4.1 Limitations

This study has several limitations. First, it is a retrospective technical report of only two highly selected patients, without a control group and with limited follow-up. Therefore, the present findings demonstrate only the technical feasibility of this approach in selected cases and cannot establish its safety, efficacy, reproducibility, or superiority over binostril expanded EEA or transcranial approaches. Second, both tumors were small and located in the midline or paramedian region, without marked lateral extension, optic canal invasion, or vascular encasement; thus, the findings cannot be generalized to larger or more complex TSMs. Third, Patient 1 developed postoperative CSF rhinorrhea, indicating that artificial-material-only reconstruction should still be evaluated cautiously. Finally, formal postoperative automated perimetry, standardized olfactory testing, and validated sinonasal quality-of-life questionnaires were not available. Larger studies with longer follow-up and standardized functional assessment are needed to further define the indications, reproducibility, and long-term outcomes of this approach.

## 5 Conclusion

In this initial two-patient experience, the neuroendoscopic single-nostril nasoseptal approach provided a feasible endonasal corridor for selected small, centrally located tuberculum sellae meningiomas without marked lateral extension, optic canal invasion, or vascular encasement. The approach allowed tumor removal through a limited transseptal corridor while preserving the middle turbinates and most intranasal structures. The use of a soft-channel system may further improve instrument maneuverability within the narrow single-nostril workspace. However, this approach should be regarded as a complementary endonasal strategy after strict case selection rather than a replacement for contemporary binostril expanded EEA or transcranial approaches. The artificial-material-only reconstruction strategy also requires cautious interpretation, particularly because postoperative CSF rhinorrhea occurred in one patient. Larger studies with longer follow-up and standardized visual, endocrine, and sinonasal outcome assessments are needed to further define the indications, reproducibility, and long-term outcomes of this technique.

## Data Availability

The original contributions presented in the study are included in the article/Supplementary material, further inquiries can be directed to the corresponding author.
